# Adenosine A_1_-A_2A_ Receptor Heteromer as a Possible Target for Early-Onset Parkinson's Disease

**DOI:** 10.3389/fnins.2017.00652

**Published:** 2017-11-22

**Authors:** Víctor Fernández-Dueñas, Andrea Pérez-Arévalo, Xavier Altafaj, Sergi Ferré, Francisco Ciruela

**Affiliations:** ^1^Unitat de Farmacologia, Departament Patologia i Terapèutica Experimental, Facultat de Medicina, IDIBELL, Universitat de Barcelona, L'Hospitalet de Llobregat, Barcelona, Spain; ^2^Institut de Neurociències, Universitat de Barcelona, Barcelona, Spain; ^3^Integrative Neurobiology Section, National Institute on Drug Abuse, Intramural Research Program, National Institutes of Health, Baltimore, MD, United States

**Keywords:** early-onset Parkinson's disease, adenosine A_1_ receptor, oligomer

Parkinson's disease (PD) is a progressive, neurodegenerative disorder that affects ~1% of individuals over the age of 60, which turns to 5% in subjects up to 85 years (de Lau and Breteler, 2006). On the other hand, a form of PD, called early-onset PD (EOPD), arises at an earlier age (<45; Bonifati et al., 2005; Ylikotila et al., 2015). EOPD patients generally display a slower progression of the disease and present a better response to dopaminergic treatments; however, they may finally develop a full PD symptomatology (i.e., bradykinesia, resting tremor, muscular rigidity and postural instability, drug-induced dyskinesia; Olgiati et al., 2016). The etiology of both PD and EOPD is still not completely elucidated. Thus, although genetic studies have provided some information about the main genes involved, epidemiological data showed that behavioral and environmental factors play a key role in the pathogenesis and progression of PD (Puschmann, 2013; Ascherio and Schwarzschild, 2016). Importantly, the contribution of genetic causes in EOPD has been extensively studied. For instance, mutations in PD-associated genes, such as *PRKN* (*PARK2*; MIM number 600116), *PINK1* (*PARK5*; MIM number 605909), and *DJ-1* (*PARK7*; MIM number 602533), have often been associated to autosomal-recessive forms of EOPD (Lücking et al., 2000; Bonifati et al., 2005; Olgiati et al., 2016). Recently, an Iranian research group described a new autosomal-recessive mutation in two siblings (30 and 34 years old) with consanguine parents, which was associated to EOPD (Jaberi et al., 2016). Interestingly, while both brothers did not present alterations in the main PD-related genes (i.e., *PRKN, PINK1*, and *DJ-1*), a homozygous missense mutation (c.835G > A) in the adenosine A_1_ receptor (A_1_R) gene (*ADORA1*) was found (Jaberi et al., 2016). This nucleotide point mutation in *ADORA1* involves the substitution of a highly conserved amino acid (p.Gly279Ser) within the transmembrane 7 (TM7) domain, but the functional consequences remain unknown. In contrast, it was recently determined that mutations affecting *ADORA1* gene and more particularly the missense matution ADORA1 (p.G279S), are not a common risk factor for PD in the European population, arguing against *ADORA1* as a candidate gene in PD (Blauwendraat et al., 2017). Altogether, these opposing data indicate that additional work must be done toward the elucidation of the potential contribution of *ADORA1* mutations in PD pathogenesis, and the contribution of genetic and environmental factors.

A_1_R has a widespread distribution in the brain, with the highest levels detected in the cortex, hippocampus, and cerebellum (Sebastião and Ribeiro, 2009). In addition, A_1_R is markedly expressed in the basal ganglia. Thus, A_1_R can be found in the major striatal neuronal population, the GABAergic medium-sized spiny neurons (MSNs; Ferré et al., 1996), together with the expression in the cortico-thalamic glutamatergic afferent fibers. These fibers, together with the dopaminergic projections from the substantia nigra pars compacta control the striatal circuitry that are critical in the control of the motor function (Sebastião and Ribeiro, 2009). The selective death of dopaminergic fibers is the primary cause and a hallmark of PD; however, the dysregulation of cortico-thalamic glutamatergic signaling is also involved in the progression of the disease (Fredholm et al., 2005; Gomes et al., 2011). Under physiological conditions, GABAergic MSNs are continuously activated by cortico-thalamic glutamatergic terminals, but a complex array of presynaptic receptors, which include A_1_R, adenosine A_2A_ receptor (A_2A_R), cannabinoid CB_1_ receptor (CB_1_R) and dopamine D_2_ and D_4_ receptors (D_2_R and D_4_R, respectively) modulate this tonic stimulation (Ciruela et al., 2006a; González et al., 2012; Mathur and Lovinger, 2012; Ferreira et al., 2015; Bonaventura et al., 2017). Potentially, the dysregulation of these presynaptic modulatory receptors can lead to abnormal glutamate release in the synaptic cleft, which may over activate postsynaptic glutamate receptors, trigger excitotoxicity and, ultimately, lead to neurodegenerative processes affecting brain circuits involved in the control of motor function (Gomes et al., 2011).

Interestingly, A_1_R colocalizes and interacts with A_2A_R at the presynaptic membrane of cortico-thalamic glutamatergic terminals, forming functional receptor heteromers in the striatum (Figure 1; Ciruela et al., 2006a). Importantly, the striatal A_1_R/A_2A_R heteromer plays a pivotal role controlling glutamate release, thus acting as an adenosine concentration-dependent switch (Ciruela et al., 2006b; Figure 1). Hence, low to moderate extracellular adenosine concentrations (homeostatic basal levels) mostly stimulate A_1_R, since it displays higher affinity for adenosine compared to A_2A_R, and a net inhibition of glutamate release is achieved (Figure 1). Conversely, moderate to high concentrations of striatal adenosine, which should theoretically trigger, in theory, both A_1_R and A_2A_R activation, ultimately lead to a predominant A_2A_R activation. In such way, A_2A_R may block heteromeric A_1_R through a receptor-receptor allosteric trans-inhibition, thus leading to a predominant facilitation of glutamate release (Figure 1; Ciruela et al., 2006b). At this point, the question consists of whether the *ADORA1* (p.G279S) mutation abolishes A_1_R function and whether this alteration depends on its heteromerization with A_2A_R receptor, specifically disrupting the function of the adenosine concentration-dependent switch. In the absence of experimental data, we can speculate that the mutation can be affecting the A_1_R/A_2A_R heteromer, resulting in a potential alteration of the fine-tuning modulation of striatal glutamatergic neurotransmission. Indeed, in such scenario, we can hypothesize that moderate concentrations of striatal adenosine would facilitate glutamate release and reduce the excitotoxicity threshold (Figure 1).

**Figure 1:**
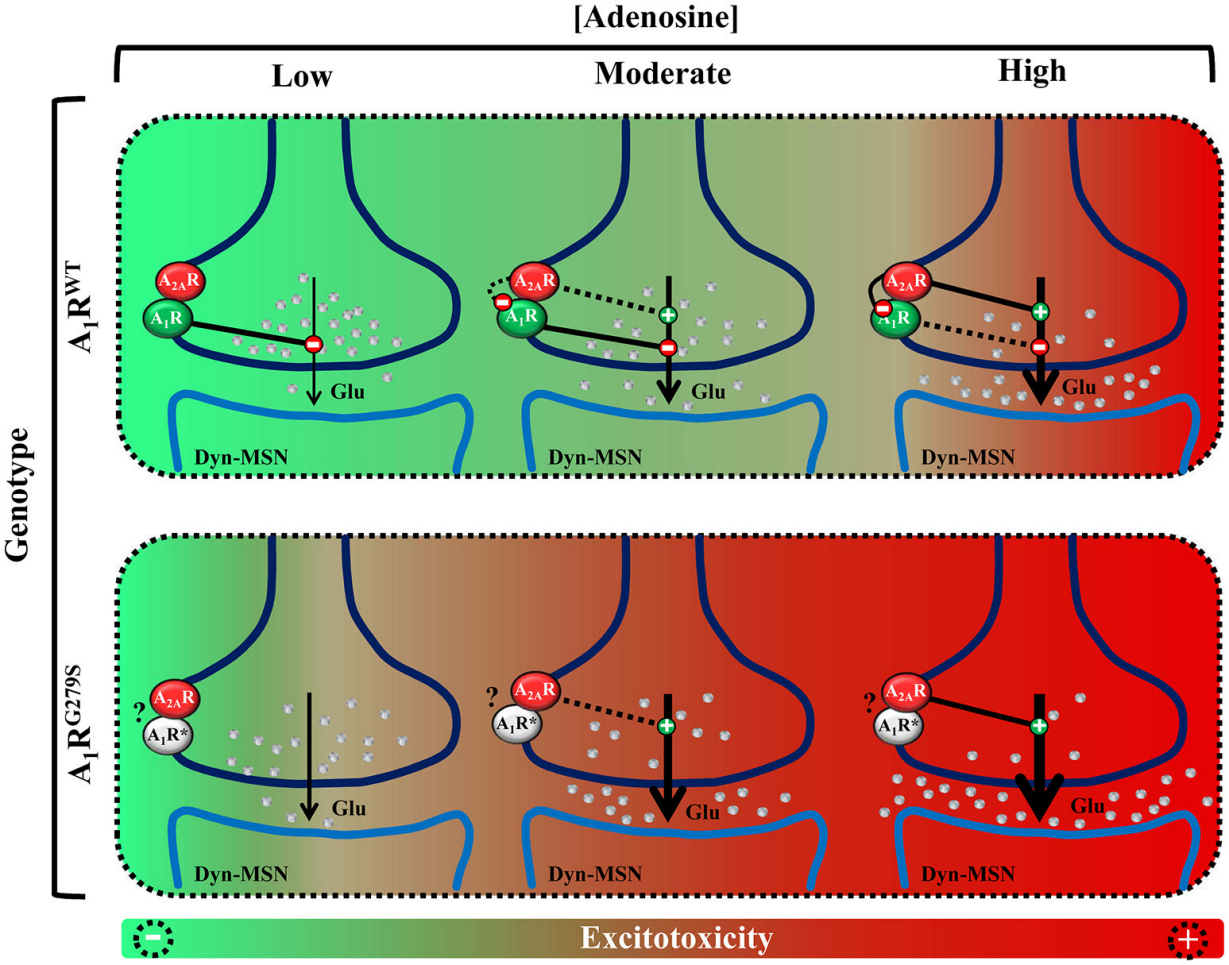
Schematic representation of the potential impact of A_1_R mutation in the fine-tuning modulation of striatal glutamatergic neurotransmission. **(Up)** Model of glutamate release control by the A_1_R/A_2A_R heteromer adenosine concentration-dependent switch. Low to moderate concentrations of adenosine activate predominantly A_1_R, inhibiting glutamate release. Moderate to high concentrations of adenosine also activate A_2A_R which, by means of the A_1_R–A_2A_R intramembrane interaction, antagonizes A_1_R function, therefore facilitating glutamate release. **(Bottom)** Model of ADORA1(p.G279S) mutation pathogenic impact (A_1_R^G279S^ or A_1_R^*^) in the striatal glutamatergic neurotransmission. The proposed A_1_R mutant loss-of-function would implicate a dysregulation of the adenosinergic presynaptic control of striatal glutamate release, which may ultimately lead to a higher risk of inducing excitotoxicity and neurodegeneration.

The dysregulation of this presynaptic module may lead to uncontrolled glutamate release which, in addition, might be potentiated by low dopamine innervation, which would not act upon inhibitory presynaptic D_2_R and D_4_R. Consequently, managing the disturbance of the adenosine switch mechanism regulating glutamatergic striatal innervation (caused either by a direct *ADORA1* mutation or mutations affecting A_1_R/A_2A_R heteromers function), may help to restore the normal functioning of the basal ganglia. In this sense, the A_1_R/A_2A_R heteromer could be considered as a potential therapeutic target for EOPD. Alternatively, the glutamatergic component of these forms of EOPD would represent an initial or master pathogenic event to dopamine denervation, as proposed in Hungtinton's disease pathophysiology (Gomes et al., 2011). In such way, the predominant role of an aberrant glutamatergic signaling could explain at the molecular level the high effectiveness of PD dopamine-based therapies, either in terms of higher or long-lasting efficacy. Nevertheless, in order to restore physiological neurotransmission it would be necessary to focus not exclusively on dopamine availability, but also in the control of glutamate release which is partially modulated by the A_1_R/A_2A_R oligomer (i.e., A_1_R activation and A_2A_R inhibition). In this sense, the use of A_2A_R antagonists has been assessed for the treatment of PD (Vallano et al., 2011). Regarding the potential use of A_1_R-based therapies, there is a major hurdle related to the A_1_R ubiquitous expression pattern that might lead to deleterious side-effects. In order to bypass these limitations, novel approaches based on (i) local A_1_R activation or (ii) pharmacological increase of the adenosine tone (below the threshold of A_2A_R activation) using adenosine transporters blockers and/or metabolizing enzymes, are expected to reach an effective treatment for EOPD.

Overall, the discovery of a novel mutation in *ADORA1* presumably leading to EOPD supports the potential beneficial use of a multimodal approach for the pharmacological treatment of this neurodegenerative condition. This approach, based on the combination of pharmacological therapies (i.e., dopaminergic compounds and drugs targeting the A_1_R/A_2A_R oligomer) could be potentially extended to all forms of PD.

## Author contributions

VF-D, XA, SF: wrote the paper; AP-A: conceived the idea; FC: conceived the idea and wrote the paper.

### Conflict of interest statement

The authors declare that the research was conducted in the absence of any commercial or financial relationships that could be construed as a potential conflict of interest.
